# Evidence that GTP-binding domain but not catalytic domain of transglutaminase 2 is essential for epithelial-to-mesenchymal transition in mammary epithelial cells

**DOI:** 10.1186/bcr3085

**Published:** 2012-01-06

**Authors:** Anupam Kumar, Jia Xu, Bokyung Sung, Santosh Kumar, Dihua Yu, Bharat B Aggarwal, Kapil Mehta

**Affiliations:** 1 Department of Experimental Therapeutics, The University of Texas M. D. Anderson Cancer Center, 1901 East Road, 4SCR3.1006, Houston, TX 77030, USA; 2 Departments of Molecular and Cellular Oncology, The University of Texas MD Anderson Cancer Center, 1515 Holcombe Boulevard, Houston, TX 77030, USA; 3 Graduate School of Biomedical Sciences, The University of Texas Health Science Center, PO Box 20334, Houston, TX 77225-0334, USA

## Abstract

**Introduction:**

The expression of proinflammatory protein tissue transglutaminase 2 (TG2) is frequently upregulated in multiple cancer cell types. However, the exact role of TG2 in cancer cells is not well-understood. We recently initiated studies to determine the significance of TG2 in cancer cells and observed that sustained expression of TG2 resulted in epithelial-to-mesenchymal transition (EMT) and promoted cancer stem cell (CSC) traits in mammary epithelial cells. These results suggested that TG2 could serve as a promising therapeutic target for overcoming chemoresistance and inhibiting metastatic spread of cancer cells.

**Methods:**

Using various mutant constructs, we analyzed the activity of TG2 that is essential for promoting the EMT-CSC phenotype.

**Results:**

Our results suggest that catalytically inactive TG2 (TG2-C277S) is as effective as wild-type TG2 (TG2-WT) in inducing the EMT-CSC in mammary epithelial cells. In contrast, overexpression of a GTP-binding-deficient mutant (TG2-R580A) was completely incompetent in this regard. Moreover, TG2-dependent activation of the proinflammatory transcription factor NF-κB is deemed essential for promoting the EMT-CSC phenotype in mammary epithelial cells.

**Conclusions:**

Our results suggest that the transamidation activity of TG2 is not essential for promoting its oncogenic functions and provide a strong rationale for developing small-molecule inhibitors to block GTP-binding pockets of TG2. Such inhibitors may have great potential for inhibiting the TG2-regulated pathways, reversing drug resistance and inhibiting the metastasis of cancer cells.

## Introduction

Despite significant advances in early detection and treatment of breast cancer, mortality due to metastatic disease remains high. A growing body of evidence supports the notion that acquisition of epithelial-to-mesenchymal transition (EMT) by breast cancer cells is an important mechanism in the progression and pathogenesis of cancer [1,2]. EMT is a developmentally regulated process in which adherent epithelial cells lose their epithelial characteristics and acquire mesenchymal properties, including fibroid morphology, characteristic changes in gene expression and increased invasion and resistance to chemotherapy [3]. In addition to eliciting the invasive phenotype, EMT also induces cancer stem cell (CSC)-like traits that are considered to provide cancer cells with the ability to self-renew and colonize at metastatic sites [4]. Thus aberrant expression of EMT regulators in breast cancer cells may contribute to disease progression, and their identification could yield novel therapeutic targets for improved patient outcomes.

In our quest to determine the significance of elevated tissue transglutaminase 2 (TG2) expression in drug-resistant and metastatic breast cancer cells [5,6], we found that stable expression of TG2 in mammary epithelial cells is associated with EMT. TG2-induced EMT was associated with constitutive activation of the NF-κB and increased expression of transcription repressors such as *Snail1*, *Twist1*, *Zeb1 *and *Zeb2 *[7]. The TG2-induced EMT is related to TGF-β signaling in that cells transfected with TG2-shRNA prior to TGF-β treatment failed to undergo EMT compared with control shRNA-transfected cells, which showed morphologic and molecular alterations typical of mesenchymal cells in response to TGF-β treatment. Importantly, TG2-induced EMT was associated with enrichment of the CD44^high^/CD24^-/low ^cell population, increased ability to form mammospheres and self-renewal ability [8], traits that are considered to endorse the CSC phenotype. These observations revealed a novel function for TG2 and suggested that TG2-regulated pathways play an important role in acquisition of drug resistance and metastasis by conferring the EMT-CSC phenotype in mammary epithelial cells.

TG2 is structurally and functionally a complex protein that has been implicated in diverse processes such as inflammation, wound-healing, celiac disease and cancer [9,10]. In addition to catalyzing calcium-dependent transamidation reactions, TG2 can bind and hydrolyze GTP. Under physiological conditions, low calcium and high GTP levels sustain TG2 in a latent form with respect to transamidation activity. Under pathological conditions, however, perturbation in calcium homeostasis and decreased GTP reserves could activate TG2 to its transamidation configuration. Researchers in several recent studies have demonstrated increased expression of TG2 in multiple cancer cell types [11-15]. Importantly, TG2 expression in cancer cells has been associated with increased resistance to chemotherapy, metastasis and poor patient outcomes [5,13,14]. Inhibition of TG2 by siRNA, antisense RNA or small-molecule inhibitors reversed the sensitivity of cancer cells to chemotherapeutic drugs and attenuated their invasion, both *in vitro *and in animal models [6,12-14,16][17]. In view of these observations, we initiated studies to determine which of the two well-characterized activities of TG2 (protein cross-linking activity and GTP-binding activity) is responsible for promoting the oncogenic functions. Herein we provide evidence that, similar to wild-type TG2, expression of transamidation-inactive mutants (C277S and W241A) is able to induce EMT-CSC in mammary epithelial cells. In contrast, the expression of the GTP-binding-deficient TG2 mutant (R580A) failed to induce EMT-CSC-related changes. Our current studies suggest that cancer cells utilize the GTP-binding and GTP-signaling function of TG2 to acquire chemoresistance and the metastatic phenotype and that this information can be exploited to develop small-molecule inhibitors to inhibit TG2-regulated pathways and reverse the EMT and CSC phenotypes.

## Materials and methods

### Materials

Immortalized human mammary epithelial cells (MCF10A) were maintained as previously described [7]. Wild-type (*WT*) and mutant TG2 constructs (*C277S*, *W241A *and *R580A*) were stably expressed in MCF10A cells by retroviral transfection and selection against puromycin as previously described [7]. Multiple stable clones were used to eliminate potential clonal effects. All experiments were performed between passages 5 and 20. Detailed procedures for lentivirus production and infection are described in Additional file 1.

### *In situ *transglutaminase activity and GTP-agarose pull-down assay

*In situ *transglutaminase activity of different TG2 constructs was determined by 5-(biotinamido)pentylamine (BPA) (Pierce Biotechnology, Rockford, IL, USA) conjugation to cellular proteins as previously described [11]. Briefly, cells were plated in six-well plates and incubated with 1 mM BPA overnight, followed by 8 hours of treatment with A23187 calcium ionophore (4 μM) to induce cytosolic calcium and TG2 activation. At the end of the incubation period, cells were lysed and equal amounts of proteins were fractionated by SDS-PAGE. Proteins were transferred onto nitrocellulose membranes and probed with horseradish peroxidase-conjugated streptavidin (GE Healthcare Life Sciences, Piscataway, NJ, USA). The membranes were stripped and reprobed with anti-TG2 mAb (CUB7401; NeoMarkers, Fremont, CA, USA) or β-actin antibodies to establish TG2 expression or even protein loading, respectively.

The GTP-agarose pull-down assay was performed to check the GTP-binding ability of different TG2 constructs according to a procedure described previously [18]. Briefly, cells were rinsed in ice-cold PBS and collected in GTP-binding buffer (20 mM Tris·HCl, pH 7.5, 5 mM MgCl_2_, 2 mM phenylmethylsulfonylfluoride, 20 mg/ml leupeptin, 20 mg/ml pepstatin, 10 mg/ml aprotinin, 300 mM NaCl and 0.5% Triton X-100). Samples were sonicated for 15 seconds and centrifuged (13,000 *g *for 10 min) at 4°C. The supernatants were collected, and 100 μg of lysate protein were incubated with 100 μl of GTP-agarose beads (Sigma-Aldrich, St Louis, MO, USA) for 30 minutes at 4°C in a 500-μl total volume of GTP-binding buffer. The beads were centrifuged at 10,000 *g *for 2 minutes, and the supernatant was retained. The beads were washed three times with 1 ml of GTP-binding buffer, and the retained supernatant was incubated with the beads overnight at 4°C. The beads were washed again as described above, and bound proteins were eluted by boiling the beads in 50 μl of 2× reducing sample buffer. The GTP-agarose-bound TG2 was visualized by immunoblotting of the eluted proteins.

### Western blotting and immunofluorescence

For Western blots, cells were lysed on ice in 50 mM Tris·HCl buffer, pH 7.5, containing 150 mM NaCl and 0.5% Nonidet P-40. Fifty micrograms of total protein from each sample were resolved on a 4% to 12% SDS bis-tris polyacrylamide gel with running buffer and transferred onto nitrocellulose membranes. The membranes were probed with various antibodies listed in Additional file 2. Immunofluorescent staining of cells in monolayer and three-dimensional cultures was performed as previously described [7].

### RT-PCR and NF-κB knockdown

The detailed procedures for RNA extraction and RT-PCR are described in Additional file 3, and primer sequences used for PCR are given in Additional file 4. Quantitative RT-PCR for EMT-associated genes was performed using the EMT-PCR Array (PAHS-090A-12; SABiosciences/QIAGEN, Frederick, MD, USA) according to the manufacturer's protocol. Expression of the p65 subunit of NF-κB was knocked down using two different p65 siRNA sequences (Cell Signaling Technology, Danvers, MA, USA) as previously described [6].

### Cell migration, invasion, colony formation, cell viability and NF-κB activity

Invasion using a Matrigel transwell assay [6], cell viability using an MTS (3-(4,5-dimethylthiazol-2-yl)-5-(3-carboxymethoxyphenyl)-2-(4-sulfophenyl)-2H-tetrazolium) assay [19] and NF-κB activity using an electrophoretic mobility shift assay [20] were analyzed as previously described. A soft agar assay also was performed as described previously [7]. Cultures were photographed, and the colonies with diameters larger than 500 mm were counted.

### Wound-healing assay

Cells were seeded in 12-well plates to about 70% to 80% confluence. A 1-ml pipette tip was used to make a horizontal scratch across the monolayer. Plates were washed to remove detached cells and were replenished with fresh medium. Wells containing scratched monolayers were then photographed under a bright-field microscope immediately before (0 hours) and after 6 to 8 hours' incubation.

### Mammosphere culture and differentiation

Mammosphere culture and differentiation were performed as described by Dontu *et al. *[21] and modified by Kumar *et al. *[8]. Briefly, after 7 to 10 days of culture in methylcellulose-containing (0.9% vol/vol to prevent cell aggregation) MammoCult medium (Stemcell Technologies, Vancouver, BC, Canada), the generated mammospheres with diameters greater than 75 μm were counted. For serial passages, mammospheres were collected by gentle centrifugation (800 rpm) and dissociated enzymatically (10 minutes in 0.05% trypsin containing 0.53 mM ethylenediaminetetraacetic acid) and mechanically using a fire-polished Pasteur pipette as described elsewhere. For differentiation, individual mammospheres were grown for 10 to 12 days in three-dimensional Matrigel culture in the presence of prolactin (2 μg/ml).

## Results

### Transglutaminase 2 expression in MCF10A cells

**Figure 1:**
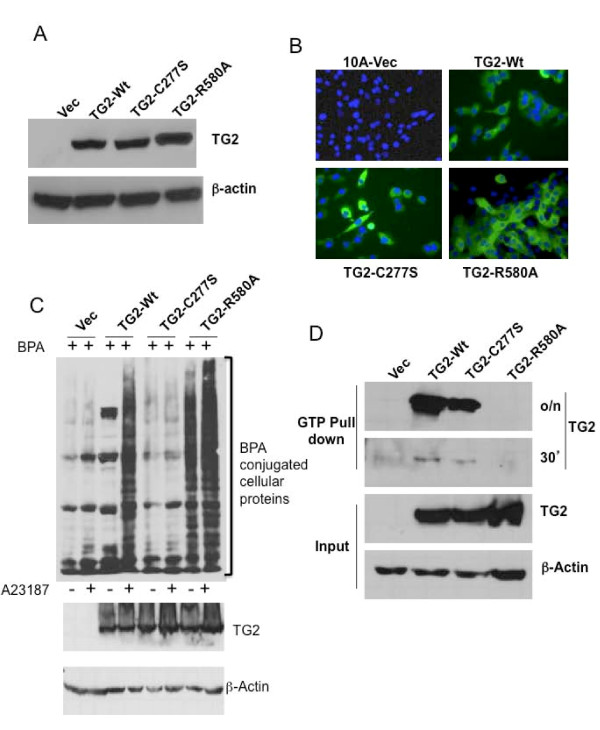
**Expression and characterization of transglutaminase 2 constructs in MCF10A cells. (A) **Representative immunoblot of cell lysates showing transglutaminase 2 (TG2) expression in MCF10A sublines stably transfected with lentiviral vector alone (Vec) or lentiviral vector containing the wild-type (TG2-WT), transamidase-inactive (TG2-C277S) or GTP-binding inactive (TG2-R580A) TG2 construct. The membrane was stripped and reprobed with anti-actin antibody to ensure even protein loading. **(B) **Immunofluorescent staining for TG2 and 4',6-diamidino-2-phenylindole (blue) staining for the nuclei in the indicated MCF10A sublines. Original magnification ×200. **(C) ***In situ *transamidation activity of TG2 constructs was studied by preincubating the indicated MCF10A cells with 1 mM 5-(biotinamido)pentylamine (BPA) overnight, followed by 8 hours of incubation in medium alone (-) or medium containing 8 μM (+) calcium ionophore, A23187. Cells were harvested, and BPA-conjugated cellular proteins were detected by an immunoblotting type assay using horseradish peroxidase-conjugated streptavidin as a probe. The membrane was reprobed with anti-TG2 and β-actin antibody to ensure even loading and levels of TG2 expression. **(D) **Immunoblot showing GTP-binding ability of different TG2 constructs. Pull-down experiments were performed with GTP-agarose beads incubated for either 30 minutes (30') or overnight (o/n) to evaluate GTP-binding ability for the indicated TG2 forms. Input shows the level of TG2 expression in MCF10A cell lines expressing different TG2 constructs.

The expression of various TG2 constructs in MCF10A cells was determined by immunoblotting (Figure 1A). MCF10A cells transfected with wild-type TG2 (TG2-WT) served as a positive control. All TG2 proteins, the TG2-WT as well as the mutant TG2 lacking transamidation activity (TG2-C277S and TG2-W241A) or GTP-binding activity (TG2-R580A), migrated as a single 78 kDa band. The level of TG2 expression was slightly higher in TG2-R580A-transfected MCF10A cells than in the TG2-C277S- or TG2-WT-transfected cells. No detectable expression of TG2 was observed in vector-transfected cells (Vec). In parallel with the immunoblotting results, we also observed the expression of all three forms of TG2 by immunostaining assay (Figure 1B). To determine the transglutaminase activity of different TG2 constructs, *in situ *transamidation activity was determined by studying the conjugation of BPA to cellular proteins. The calcium ionophore A23187 was used to induce intracellular calcium and activation of TG2. As expected, no BPA conjugation was evident in cells lacking TG2 expression (Vec) or harboring catalytically inactive mutant protein (TG2-C277S), whereas TG2-WT- and TG2-R580A-transfected cells showed significant conjugation of BPA to multiple cellular proteins. Both the basal and A23187-induced conjugation of BPA were much higher in TG2-R580A cells (Figure 1C). These results suggest that TG2-R580A cells have constitutive active transglutaminase activity due to the lack of GTP-binding function, whereas TG2-C277S cells are deficient in transglutamination activity even in the presence of calcium. To determine the GTP-binding ability of different TG2 constructs, we performed the GTP agarose pull-down assay. We confirmed that only TG2-WT and TG2-C277S bind to GTP-agarose (Figure 1D). As previously observed [18], the extent of GTP binding to TG2-C277S was relatively less than to TG2-WT. TG2-R580A failed to bind GTP agarose, even after overnight incubation. These results suggest that the GTP-binding ability of TG2 proteins is TG2-WT > TG2-C277S > TG2-R580A, in that order.

### GTP-binding function of transglutaminase 2 is essential for inducing mesenchymal transition

**Figure 2:**
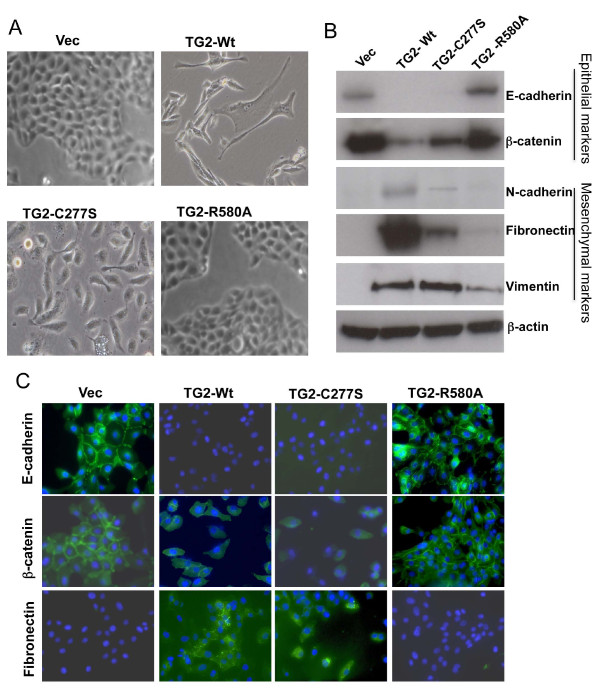
**GTP-binding activity is critical for transglutaminase 2-induced epithelial-to-mesenchymal transition**. **(A) **Phase-contrast images of MCF-10A cells expressing the lentiviral vector alone (Vec) or vector containing wild-type (TG2-WT) or mutant transglutaminase 2 (TG2) constructs (TG2-C277S and TG2-R580A) were taken after 48 hours of culture in puromycin selection medium. Original magnification ×200. **(B) **Immunoblot analysis of control (Vec) and TG2-expressing MCF10A cells. Expression of epithelial cell markers (E-cadherin and β-catenin) and mesenchymal cell markers (N-cadherin, fibronectin and vimentin) was examined by immunoblotting. **(C) **Immunofluorescence due to expression of epithelial-to-mesenchymal transition markers in MCF10A sublines. Cells were counterstained with 4',6-diamidino-2-phenylindole (blue) for nuclear staining.

We have previously observed that neoexpression of TG2 results in induction of EMT in nontransformed MCF10A and MCF12A mammary epithelial cells [7]. Similarly to the TG2-WT cells, TG2-C277S cells acquired spindle-shaped morphology and exhibited scattered distribution with a fibroblast-like appearance (Figure 2A). In contrast, the TG2-R580A cells appeared similar to the control vector-transfected cells, with a cobblestone-like epithelial appearance and tight cell-to-cell junctions (Figure 2A). These observations suggest that the GTP-binding activity of TG2 might be essential for inducing EMT in MCF10A cells. To test this notion, we next studied various EMT-related molecular alterations in TG2-transfected cells. We found that TG2-R580A cells expressed high basal levels of epithelial marker proteins (E-cadherin and β-catenin) with minimal or undetectable alterations in mesenchymal marker proteins (fibronectin, vimentin and N-cadherin) (Figure 2B). The TG2-WT and TG2-C277S cells, in contrast, showed almost a complete loss of E-cadherin, decreased β-catenin and increased expression of mesenchymal markers (N-cadherin, fibronectin and vimentin) (Figure 2B). These changes were further confirmed by immunofluorescent staining. The vector-infected and TG2-R580A cells showed distinct membranous staining for E-cadherin and β-catenin and diminished staining for fibronectin. In contrast, TG2-WT and TG2-C277S cells showed no E-cadherin staining, decreased β-catenin staining and increased fibronectin staining (Figure 2C). Overall, these results suggest that aberrant expression of WT or catalytically inactive (C277S) TG2 mutants is effective in promoting the EMT and that the GTP-binding function of TG2 is critical to this effect.

### GTP-binding activity of transglutaminase 2 is essential for Snail, Twist, and Zeb expression

**Figure 3:**
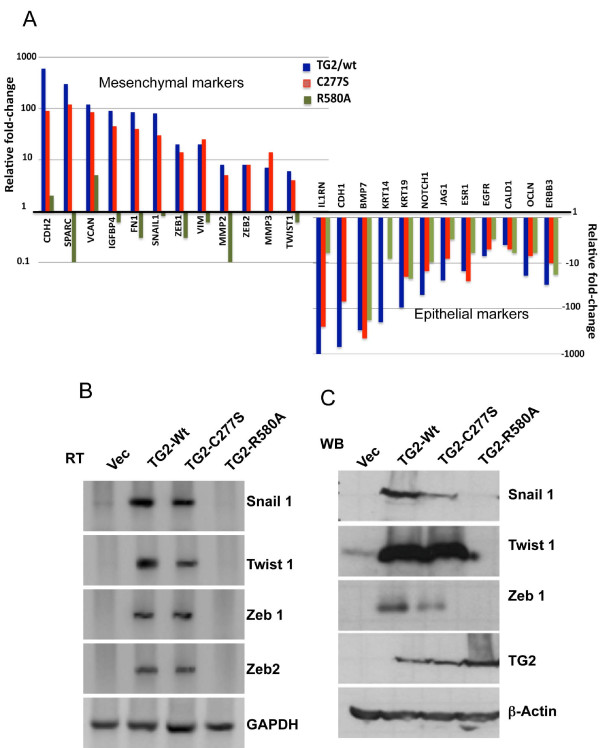
**GTP-binding inactive transglutaminase 2 is unable to induce the epithelial-to-mesenchymal transition transcription repressors**. **(A) **Real-time RT-PCR array showing changes in the expression of epithelial-to-mesenchymal transition-related genes in MCF10A sublines expressing wild-type (WT) or mutant constructs of transglutaminase 2 (TG2) relative to control lentiviral vector (Vec)-transfected MCF10A cells. The *y*-axis denotes the fold change in transcript levels, the blue bars denote the gene expression in TG2-WT cells, the red bars denote gene expression in TG2-C277S and the green bars denote gene expression in TG2-R580A cells. The expression of glyceraldehyde 3-phosphate dehydrogenase (GAPDH), β-actin and 18S ribosomal RNA was used to normalize variable template loading. **(B) **RT-PCR analysis (RT) and **(C) **Western blot analysis (WB) were performed to validate the expression of transcription factors *Snail1*, *Zeb1 *and *Twist1 *in different MCF10A sublines.

Induction of EMT involves complex molecular alterations primarily choreographed by transcription factors such as *Snail1*, *Slug*, *Twist*, *Zeb1*, *Zeb2, E12 *and *E47 *[2,3]. To determine the effect of TG2 mutants on transcriptional regulation of EMT-associated genes, we determined the transcriptional profile of EMT-associated genes using a real-time PCR-based EMT array (SABiosciences/QIAGEN). The results shown in Figure 3A demonstrate the fold change relative to the control cells (MCF10A-vec) in EMT-associated genes in MCF10A cells transfected with different TG2 constructs. The results obtained revealed a several-fold decrease in the expression of epithelial marker genes such as *CDH1 *(E-cadherin), *KRT19 *(keratin 19), *BMP7 *(bone morphogenetic protein 7) and *OCLN *(occludin), with concomitant increases in mesenchymal marker genes such as *CDH2 *(N-cadherin), *VCAN *(versican), *FN1 *(fibronectin) and *VIM *(vimentin) in TG2-C277S and TG2-WT cells. In TG2-R580A cells, a slight decrease in epithelial marker genes (*KRT19*, *KRT14*, *CALD1 *and *OCLN*) was observed, but no change in the *CDH1 *gene transcript was evident. For mesenchymal marker genes such as *CDH2*, *VCAN*, *FN1*, *SPARC *and *VIM*, expression either was unaltered or even the basal expression of these genes was downregulated in TG2-R580A cells (Figure 3A). Overall, these results suggest that TG2-WT and TG2-C277S cells undergo mesenchymal transition, whereas TG2-R580A cells continue to exhibit an epithelial phenotype. Moreover, the expression of transcription repressors *Snail1*, *Zeb1*, *Zeb2 *and *Twist1 *in TG2-WT and TG2-C277S cells was upregulated, whereas these transcription factors were downregulated in TG2-R580A cells. Altered expression of *Snail1*, *Zeb1 *and *Twist1 *in response to WT and mutant TG2 expression was further validated by RT-PCR (Figure 3B) and Western blot analysis (Figure 3C). These results imply that transcriptional repression of E-cadherin and upregulation of fibronectin, N-cadherin and vimentin in MCF10A cells in response to TG2 expression entail intact GTP-binding activity, whereas transamidation activity is not essential for inducing these changes.

### GTP-binding activity of transglutaminase 2 is essential for promoting invasiveness and drug resistance

**Figure 4:**
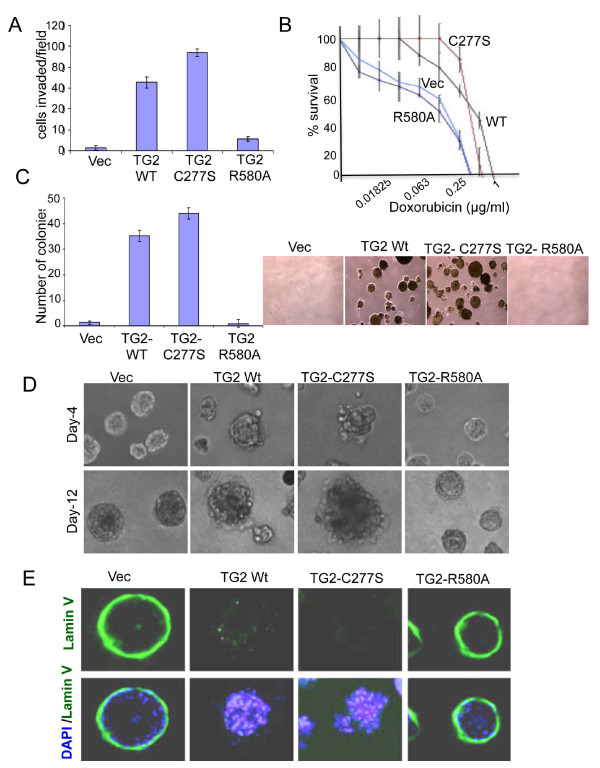
**GTP-binding deficienttransglutaminase 2 fails to promote survival, invasion and drug resistance**. **(A) **A transwell Matrigel invasion assay was performed with the indicated MCF10A sublines. Cells that invaded through the Matrigel after 72 hours of incubation were counted in five random microscopic fields. Original magnification ×20. Experiments were performed three times in triplicate. The results shown are the average number of invading cells per field ± SEM. Vec, lentiviral vector. **(B) **The sensitivity of the indicated MCF10A sublines to doxorubicin is shown. Quadruplicate wells in 96-well plates, containing 2,000 cells per well in 0.2 ml of the complete medium (10% FCS), were either left untreated or treated with the indicated concentrations of doxorubicin. Two days after the treatment viable cells remaining in the wells were determined by the 3-(4,5-dimethylthiazol-2-yl)-5-(3-carboxymethoxyphenyl)-2-(4-sulfophenyl)-2H-tetrazolium (MTS) reduction test, and percentage cell viability was calculated. Experiments were repeated at least three times with similar results. Bars, mean of quadruplicate values from a representative experiment; lines, SD. **(C) **Average number of colonies formed from three independent experiments ± SEM after 3 weeks of incubation of the cells in soft agar. Images of colonies formed from a representative experiment after 3 weeks of culture in soft agar are shown in the right panel. Original magnification ×10. **(D) **Phase-contrast images of acinar structures (at days 4 and 12) formed as a result of the indicated MCF10A cells cultured in Matrigel-coated glass slide chambers for the indicated time periods. **(E) **Loss of basement membrane in MCF10A sublines expressing WT- or C277S-TG2 after 12 days of culture in Matrigel-coated chambers. Structures thus obtained were immunostained for laminin V (green) and 4',6-diamidino-2-phenylindole (blue). Images shown are from a representative experiment repeated twice with similar results. Original magnification ×20.

Metastasis is a multistep process and involves increased migration, protease secretion and altered adhesion of cancer cells to allow their dissemination from primary tumor sites [22]. In this context, our finding that stable expression of TG2-WT or TG2-C277S in MCF10A cells is associated with increased expression of the matrix metalloproteases MMP2 and MMP3 (Figure 3A) and transition to the mesenchymal state (Figure 2A), whereas TG2-R580A expression results in decreased MMP2 levels (Figure 3A) without any change in cellular morphology, is of interest. Moreover, the TG2-C277S cells showed increased motility in the wound-healing assay, whereas TG2-WT and TG2-R580A cells showed low motility, similarly to the 10A-Vec cells (Additional file 5). To further evaluate the invasive potential, we performed a Matrigel invasion assay and found that the number of cells that invaded through Matrigel was significantly higher for TG2-WT and TG2-C277S cells than for the vector-infected or TG2-R580A cells (Figure 4A). These results further support our hypothesis that catalytic inactive TG2 (C277S) is as effective as WT-TG2 in inducing EMT and promoting an invasive phenotype in epithelial cells, whereas GTP-binding-deficient (R580A) TG2 lacked this ability.

Next we determined the effect of different TG2 forms in conferring resistance to doxorubicin. The results shown in Figure 4B reveal that cells expressing TG2-C277S and TG2-WT are relatively resistant to doxorubicin-induced killing compared with TG2-R580A or vector-transfected cells. After that step, we determined the oncogenic potential of three TG2 constructs by studying anchorage-independent growth of cells in soft agar, an *in vitro *surrogate measure for tumorigenicity [23]. We found that catalytically inactive TG2-expressing MCF10A cells (TG2-C277S) showed high frequency of colony formation, similar to that for TG2-WT cells, whereas GTP-binding-defective TG2-R580A cells, similarly to the control MCF10A-Vec cells, failed to survive and form colonies under these conditions (Figure 4C).

Previously, we reported that TG2 expression in MCF10A cells disrupts their normal organization into acinar structures when grown in three-dimensional culture [7]. We next sought to determine whether catalytic inactive mutants (TG2-C277S) or GTP-binding-null mutants (TG2-R580A) would have a similar effect on acinar organization. We found that TG2-R580A cells, similarly to vector-infected cells, formed well-organized acinar structures (Figure 4D). These acinar structures contained hollow lumina with laminin V staining all around the basement layer, demonstrating apicobasal polarization to the cells (Figure 4E). Moreover, the vector-infected and TG2-R580A cells generated acinar spheroids that showed strong E-cadherin expression in cells adjacent to the basal layer (Additional file 6). In contrast, TG2-C277S and TG2-WT cells produced acinar spheroids with disrupted architecture. They were relatively larger in size without the lumen (Figure 4D). Moreover, these structures showed diffused laminin staining in the basement membrane (Figure 4E), and the cells lacked E-cadherin expression (Additional file 6). These results suggest that the GTP-binding function of TG2 is critical for promoting cell motility, invasiveness and anchorage-independent growth in mammary epithelial cells.

### GTP-binding function of transglutaminase 2 is essential for NF-κB activation

**Figure 5:**
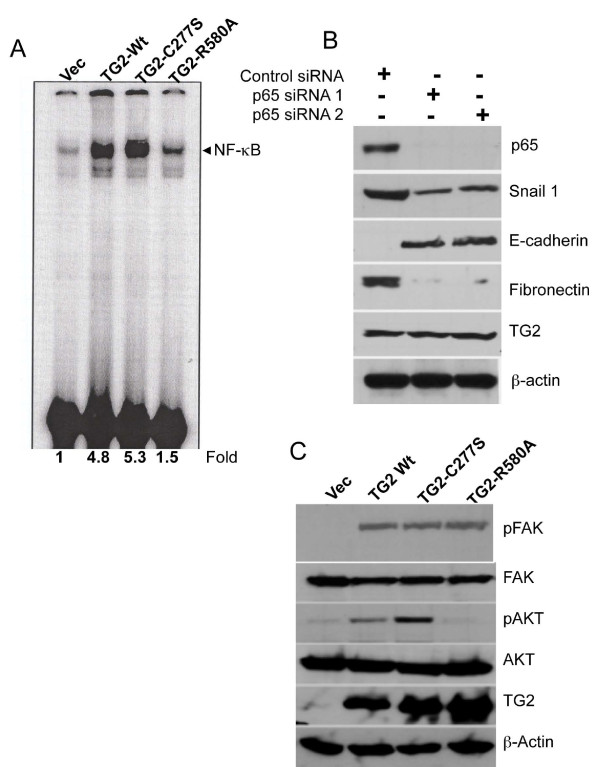
**Transglutaminase 2 requires a functional GTP-binding domain to activate NF-κB and Akt signaling**. **(A) **Electrophoretic mobility shift assay for NF-κB activity in the nuclear extracts prepared from the indicated MCF-10A cells. **(B) **Immunoblot analysis for different epithelial and mesenchymal marker proteins in transglutaminase 2 wild-type (TG2-WT) cells before and after transfection with either control or p65-specific siRNA. **(C) **Immunoblot analysis showing the levels of total and pAKT (S473) and phosphorylated focal adhesion kinase (pFAK) (Y397) in control and TG2-transfected MCF10A sublines.

TG2 expression in normal and transformed cells is associated with constitutive activation of NF-κB [20,24], the transcription factor that has been implicated in EMT [25,26]. Therefore, we next sought to determine whether failure of the TG2-R580A mutant to induce EMT is related to its NF-κB regulatory function. Our results (Figure 5A) confirm earlier observations that TG2-WT expression is associated with constitutive NF-κB activity in MCF10A cells. Importantly, the catalytically inactive TG2-C277S was as effective as TG2-WT in inducing NF-κB activation, whereas the GTP-binding-null TG2-R580A mutant was considerably less active in this regard. To determine the significance of TG2-induced NF-κB activity in the EMT process, we knocked down the expression of the p65 subunit of NF-κB in TG2-expressing MCF10A cells and determined various epithelial and mesenchymal markers. The results indicated that downregulation of p65 expression in TG2-WT cells could dramatically reverse the EMT phenotype (MET) without affecting TG2 levels (Figure 5B). Hence, knockdown of the p65 subunit reversed the expression of *Snail1*, increased the expression of the epithelial marker E-cadherin and reduced the expression of the mesenchymal marker fibronectin without affecting TG2 expression (Figure 5B). These results establish that NF-κB activation is an important downstream mediator of TG2-induced EMT. Similarly, other mediators, such as activated Akt and focal adhesion kinase (FAK), which are known to support the transition of epithelial cells to the mesenchymal state, were also found to be activated in TG2-expressing cells. Akt activation was selectively observed in TG2-WT and TG2-C277S cells, however, whereas FAK activation was evident in all three types of TG2-expressing cells (Figure 5C).

### GTP-binding activity and stem cellness

**Figure 6:**
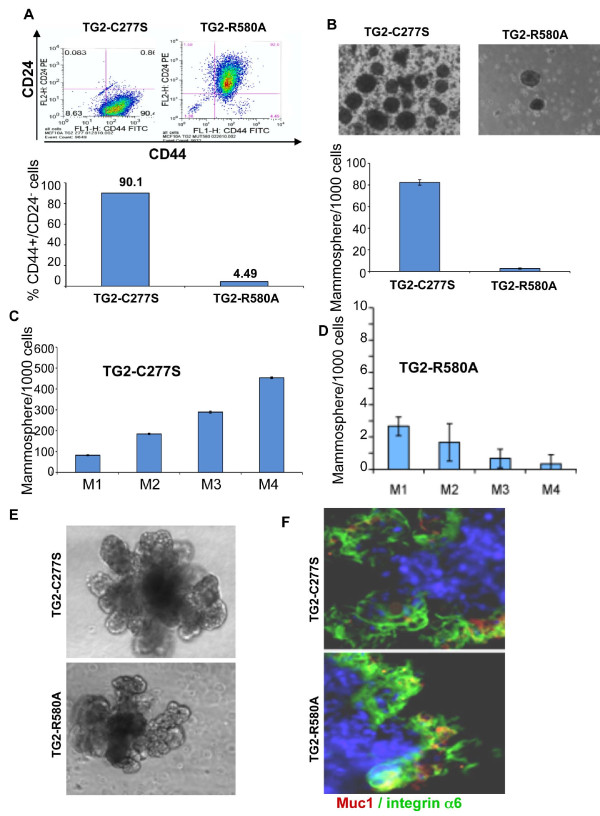
**GTP-binding function of transglutaminase 2 is essential for promoting the stem cell phenotype**. **(A) **Fluorescence-activated cell sorting analysis for the mammary stem cell antigenic markers CD44 and CD24 in MCF10A cells expressing catalytically inactive (TG2-C277S) and GTP-binding-defective (TG2-R580A) forms of transglutaminase 2 (TG2). **(B) **Phase-contrast images of mammospheres formed by the indicated MCF10A cells (upper panel). *In vitro *quantification of mammospheres formed by TG2-C277S- and TG2-R580A-transfected MCF-10A cells (lower panel). The data shown are the number of mammospheres formed per 1,000 seeded cells ± SEM. **(C) ***In vitro *quantification of mammospheres formed by TG2-C277S-expressing MCF10A cells at each serial passage (M1 to M4). **(D) ***In vitro *quantification of mammospheres formed by TG2-R580A-transfected MCF10A cells after different serial passages (M1 to M4). The data shown are the average number of mammospheres formed per 1,000 seeded cells ± SEM of triplicate values from a representative experiment. Note the difference in scale (600 vs 10) for (C) and (D). **(E) **Phase-contrast images of differentiated mammospheres following their 12-day culture in Matrigel in the presence of prolactin. **(F) **The differentiated structures were immunostained for luminal marker Muc1 (red), for basal marker CD49f/integrin α_6 _(green) and for nuclei with 4',6-diamidino-2-phenylindole (blue).

Next we studied whether the GTP-binding function of TG2, which seems to be critical for inducting EMT, has any effect on the acquisition of stem cell phenotype. Analysis of the antigenic mammary stem cell surface markers CD44 and CD24 and the ability to form mammospheres suggested that, although TG2-WT [8] and its catalytically inactive TG2-C277S mutant resulted in enrichment of the CD44^high^/CD24^low ^cell subpopulation (Figure 6A) and increased the ability to form mammospheres (Figure 6B), the GTP-defective TG2-R580A mutant failed to do so (Figure 6). We then tested the self-renewal capability of cells using an *in vitro *assay that relies on assessing the sphere initiation efficiency of serially passaged cells cultured as mammospheres. Once again TG2-WT [8] and TG2-C277S were equally efficient in promoting the self-renewal ability of MCF10A mammospheres (Figure 6C). In contrast, the TG2-R580A-expressing cells showed a progressive decrease in the number of mammosphere-forming cells with each passage (Figure 6D). Another important feature of stem cells is their ability to differentiate into multiple lineages. To determine how TG2 mutations could affect the ability of stem cells to differentiate into secondary structures, we transferred individual mammospheres into Matrigel cultures. Under these culture conditions, the mammospheres derived from either TG2-C277S- or TG2-R580A-expressing cells differentiated and formed complex secondary structures representing mammary gland-like organotypic outgrowths (Figure 6E). Immunostaining of these secondary structures revealed the presence of both Muc1 (luminal marker)- and CD49f/integrin α_6 _(basal marker)-positive cells in both the TG2-C277S- and TG2-R580A-transfected MCF10A secondary structures (Figure 6F). These results reflect that although the R580A mutation inhibits TG2's ability to induce EMT and to promote stem cellness in mammary epithelial cells, it does not interfere with their ability to differentiate.

## Discussion

Several reports have documented elevated expression of TG2 in multiple cancer cell types [27,28]. TG2 expression in cancer cells has been linked to drug resistance and metastasis, the phenotypes that account for nearly 90% of cancer-related deaths [29]. These observations clearly imply that TG2 could be an attractive therapeutic target for the treatment of drug-resistant and metastatic cancers. As a first step in this direction, we determined the activity of TG2 that is responsible for promoting its oncogenic functions. We employed TG2-C277S and TG2-W241A mutants (data not shown) as the transamidation-inactive forms and TG2-R580A mutant as the GTP-binding-deficient form. The GTP-binding and transamidation activities of constructs in stably transfected MCF10A cell lines were found to be consistent with predicted activities reported in the literature and based on the structure and function information of TG2 [30-32].

The GTP-binding and transamidation activities of TG2 are mechanistically mutually exclusive and are regulated by GTP and calcium. The transamidase activity of TG2 is stimulated by calcium and inhibited by GTP, whereas GTPase activity is inhibited by calcium [33]. It is not clear whether under physiological conditions intracellular TG2 acts as a transamidase or as a GTPase; however, the prevailing view is that, due to high GTP and low calcium, TG2 exists as an inactive transamidase inside the cell, where it acts mainly as a GTP-binding protein and converts GTP into GDP. This view is supported by our current results, in which TG2-WT-expressing MCF10A cells failed to show any significant *in situ *activity under basal culture conditions. The GTP-binding inactive mutant (TG2-R580A), in contrast, showed some transamidase activity even under basal conditions (Figure 1). The transamidating *in situ *activity increased in response to calcium ionophore (A23187) treatment in TG2-WT- and TG2-R580A-transfected cells, but not in transamidase-inactive TG2-C277S-expressing cells. Interestingly, the transamidation activity of TG2 had no relevance to its ability to induce EMT-CSC in mammary epithelial cells. Thus the catalytically inactive TG2-C277S and TG2-W241A forms of TG2 were as effective as TG2-WT in inducing the EMT-CSC-related changes, except for some subtle quantitative differences in the levels of some inducible genes (Figure 2). The GTP-binding-inactive R580A mutant, on the other hand, was completely inactive in inducing the EMT-CSC-related changes, despite its high expression and transamidation activity. The expression of each of the three constructs was associated with decreased growth rates in MCF10A cells and was observed to be Vec > C277S > WT > R580A, in that order (Additional file 7).

TG2 has received considerable attention in recent years for its potential role in cancer cells. There is ample evidence supporting metastatic and drug-resistant cancer cells' expression of high basal levels of TG2 [5,6,11,12,14,15,17]. Our quest to understand the significance of TG2 in cancer cells led us to some rather surprising findings. We found that stable expression of TG2 in normal and transformed mammary epithelial cells is associated with the induction of EMT [7] and CSC [8], the phenotypes that are closely linked with the development of drug resistance and metastasis in cancer cells [34,35]. These observations clearly imply that aberrant expression of TG2 in cancer cells could promote drug resistance and metastasis by inducing the EMT-CSC phenotype and hence could serve as a promising therapeutic target for reversing chemoresistance and inhibiting metastasis. Indeed, the observations that inhibition of TG2 by siRNA, small-molecule inhibitors or antisense RNA could render cancer cells sensitive to chemotherapeutic drugs and inhibit their invasiveness both *in vitro *and in animal models [13,14,17,24] strongly support such a contention. Therefore, knowledge of the TG2 domain and function that is essential for promoting the EMT-CSC is foundational for the rational design of small-molecule inhibitors that are able to harness TG2-regulated events in cancer cells.

With this intent, we initiated studies to address whether either or both of the two well-characterized activities (transamidation and GTPase) of TG2 are essential for promoting EMT-CSC in mammary epithelial cells. Our findings strongly suggest that the transamidation activity of TG2 is not essential for promoting the EMT-CSC. However, interference with GTP-binding function could completely abrogate its oncogenic functions. Whether the failure of the TG2-R580A mutant to support the EMT-CSC phenotype is related to its inability to bind and hydrolyze GTP or is a consequence of the conformational change induced as a result of single-amino acid substitution in position 580 remains to be determined. Indeed, it has been suggested that the TG2-R580A mutant acquires a more open and extended conformation compared with the TG2-WT form [31]. Hence the failure of TG2-R580A to promote EMT-CSC may be related to its altered interaction with some signaling proteins rather than to its inability to bind and hydrolyze GTP. In this context, our recent observation that the interaction of TG2 with the inhibitor of NF-κB (IκB) results in constitutive activation of NF-κB (Kumar S and Mehta K, unpublished data) is of interest. It is likely that TG2-R580A is unable to interact effectively with IκB, owing to its extended conformation. Indeed, the TG2-induced activation of NF-κB was significantly compromised in TG2-R580A-transfected cells compared with the TG2-WT, TG2-C277S (Figure 5A) or TG2-W241A cells (data not shown). Constitutive activation of NF-κB is frequently associated with advanced-stage cancers and is known to confer resistance to chemotherapy and to promote metastasis by inducing EMT [36-38]. As a proof of concept, our data support the hypothesis that TG2-induced EMT in MCF10A cells could be reversed (MET) by inhibiting NF-κB activity. Thus downregulation of the p65 subunit of NF-κB reversed the mesenchymal phenotype to the epithelial phenotype without any noticeable change in TG2 expression (Figure 5B). Recently, Shao *et al. *[25] noted similar involvement of NF-κB in TG2-induced EMT, invasiveness and drug resistance in ovarian cancer cells. Overall, these observations suggest that open or closed conformation of TG2, which depends on the intracellular environment, is the major determining factor in TG2-induced promotion of cell survival or cell death signaling. Thus transamidase-inactive closed conformation of TG2 (due to GTP binding in the presence of low calcium) may promote cell survival processes by serving as a scaffold protein and mediating protein-protein interactions. In this context, the band 4.2 protein in red blood cells, the only catalytically inactive member of the transglutaminase family of proteins, primarily serves as a scaffold protein and promotes interaction with various membrane proteins, such as ankyrin, spectrin and CD47 [39,40]. Given the structural similarities between TG2 and protein 4.2, it is tempting to speculate that the primary function of TG2 in cancer cells is to serve as a scaffold protein rather than as an enzyme. In this capacity, TG2 can promote protein-protein interactions, resulting in constitutive activation of cellular events needed for increased cell survival and invasive function during advanced-stage cancer.

## Conclusions

Our results provide evidence that TG2's aberrant expression in drug-resistant and metastatic cancer cells facilitates cell survival and invasive functions independently of its transamidation activity. Recently, Colak *et al. *[30] reached a similar conclusion, supporting the notion that conformational state rather than transamidation activity of TG2 is an important determinant in cell survival signaling under glucose-deprived conditions. These findings provide new insights into novel oncogenic functions of TG2 and offer promising leads for developing small-molecule inhibitors to intervene in TG2-regulated processes and to prevent the progression of cancer to metastatic disease.

## Abbreviations

EMT: epithelial-to-mesenchymal transition; FAK: focal adhesion kinase; FCS: fetal calf serum; GTP: guanine triphosphate; mAb: monoclonal antibody; NF-κB: nuclear factor κB; PBS: phosphate-buffered saline; RT-PCR: reverse transcriptase polymerase chain reaction; SDS-PAGE: sodium dodecyl sulfate polyacrylamide gel electrophoresis; shRNA: small hairpin RNA; siRNA: small interfering RNA; TG2: transglutaminase 2; Vec: vector; WT: wild type.

## Competing interests

The authors declare that they have no competing interests.

## Authors' contributions

AK and KM designed the experiments. AK, JX, BS and SK performed the experiments. BBA and DY contributed new reagents or analytical tools. AK and KM wrote the article. All authors read and approved the final manuscript.

## Supplementary Material

Additional file 1 **Protocol for lentivirus production and infection**. Click here for file

Additional file 2 **Table 1 Antibodies used for immunofluorescence and immunoblotting**. Click here for file

Additional file 3 **Protocol for RNA extraction, RT-PCR and quantitative RT-PCR**. Click here for file

Additional file 4 **Table 2 Primers used for PCR**. Click here for file

Additional file 5 **Supplementary Figure **1**Effect of various transglutaminase 2 constructs on cell motility**. The wound-healing assay with indicated MCF10A sublines was performed. Cell cultures were photographed at 0 and 6 hours after wounding. Click here for file

Additional file 6 **Supplementary Figure **2**Effect of various transglutaminase 2 constructs on acinar structure assembly**. MCF10A cells stably transfected with indicated construct of transglutaminase 2 (TG2) were cultured in Matrigel-coated chambers for 12 days and immunostained for E-cadherin (green), TG2 (red) and 4',6-diamidino-2-phenylindole (blue). Representative images from two independent experiments with similar results are shown. Original magnification ×20. Click here for file

Additional file 7 **Supplementary **Figure 3**Effect of various transglutaminase 2 constructs on MCF10A cell growth**. Cells (*n *= 2,000) expressing indicated form of transglutaminase 2 (TG2) were cultured in quadruplicate in a 96-well plate, and the number of cells in each well was determined after 72 hours of culture. Click here for file
