# Supplementary material for: Evidence that GTP-binding domain but not catalytic domain of transglutaminase 2 is essential for epithelial-to-mesenchymal transition in mammary epithelial cells
Source: Breast Cancer Res. 2012 Jan 6;14(1):R4. doi: 10.1186/bcr3085 (PMC3496119; doi:10.1186/bcr3085)
Supplement: Additional file 1 — Protocol for lentivirus production and infection. [file bcr3085-S1.DOCX]

**Additional file 1**

**Protocol for lentivirus production and infection**

Lentiviral vector and its packaging vectors were transfected into 293T packaging cells by lipofectamine transfection. Briefly, 293T cells were aliquoted (1x10^6^) into 25 cm^2^ flask one day before transfection. Then the cells were transfected with 2.5 μg of different TG2 constructs in pCDH vector, together with 1.875 μg of psPAX2 (packaging vector) and 0.625 μg of pMD2G (envelope vector). After 5 hr incubation, the transfection medium was replaced with fresh culture medium; 48 hr later, the lentivirus-containing medium was collected and centrifuged at 1500 rpm for 5 min to pellet the cell debris, the supernatant was passed through a 0.45-μm filter, and the target cells were infected with fresh lentivirus-containing medium (supplemented with 8 μg/ml Polybrene) for 48 hr.
