# Supplementary material for: Evidence that GTP-binding domain but not catalytic domain of transglutaminase 2 is essential for epithelial-to-mesenchymal transition in mammary epithelial cells
Source: Breast Cancer Res. 2012 Jan 6;14(1):R4. doi: 10.1186/bcr3085 (PMC3496119; doi:10.1186/bcr3085)
Supplement: Additional file 2 — Table 1 Antibodies used for immunofluorescence and immunoblotting. [file bcr3085-S2.DOCX]

| *Antigens* | *Clone/Cat #* | *Source* | *Application* |
| --- | --- | --- | --- |
| TG2 | CUB7402 | Neomarkers | IB, IFS |
| E-cadherin | Sc-21791 | Santa Cruz Biotech | IB |
| E-cadherin | 610182 | BD Transduction Laboratories | IF |
| N-cadherin | Sc-59987 | Santa Cruz Biotech | IB |
| Fibronectin | sc-9068 | Santa Cruz Biotech | IB,IFS |
| vimentin | Sc-66001 | Santa Cruz Biotech | IB,IFS |
| β-catenin | 610154 | BD Transduction Laboratories | IB,IFS |
| β-actin | A2172 | Sigma-Aldrich | IB |
| Snail1 | Sc-28199 | Santa Cruz Biotech | IB |
| Twist1 | sc-15393 | Santa Cruz Biotech | IB |
| Zeb1 | Sc-81428 | Santa Cruz Biotech | IB |
| CD49f/integrin α6 | 555734 | BD Pharmingen | IFS |
| Muc1 | 550486 | BD Pharmingen | IFS |
| pAkt | 9271 | Cell signaling technology | IB |
| Akt | 4691 | Cell signaling technology | IB |
| pFAK | 611722 | BD Transductuion laboratories | IB |
| FAK | 610087 | BD Transductuion laboratories | IB |
| Laminin V | MAB1947 | Chemicon International | IFS |

**Table 1. Antibodies used for immunofluorescence and immunoblotting**

IB: Immunoblot, IFS: Immunofluorescence staining,
