# Supplementary material for: Evidence that GTP-binding domain but not catalytic domain of transglutaminase 2 is essential for epithelial-to-mesenchymal transition in mammary epithelial cells
Source: Breast Cancer Res. 2012 Jan 6;14(1):R4. doi: 10.1186/bcr3085 (PMC3496119; doi:10.1186/bcr3085)
Supplement: Additional file 3 — Protocol for RNA extraction, RT-PCR and quantitative RT-PCR. [file bcr3085-S3.DOCX]

**Additional file 3**

**Protocol for RNA extraction, RT-PCR and quantitative RT-PCR**

Total RNA was extracted using Quiagen mini-RNA isolation kit according to the manufacturer’s protocol. For RT-PCR, 2 μg total RNA was reverse transcribed to cDNA using Superscript III First Strand Synthesis System (Invitrogen). An equivalent volume (2 μl) of cDNA was used as the template for PCR using gene-specific primers. Quantitative RT-PCR for EMT-associated genes was performed, using SAbiosciences EMT array. Relative change was calculated after normalization to GAPDH, β actin and 18s ribosomal RNA
