# Supplementary material for: Evidence that GTP-binding domain but not catalytic domain of transglutaminase 2 is essential for epithelial-to-mesenchymal transition in mammary epithelial cells
Source: Breast Cancer Res. 2012 Jan 6;14(1):R4. doi: 10.1186/bcr3085 (PMC3496119; doi:10.1186/bcr3085)
Supplement: Additional file 4 — Table 2 Primers used for PCR. [file bcr3085-S4.DOCX]

**Table 2 – Primers used for PCR**

| Gene | Primers |
| --- | --- |
| *E-cadherin* | F: 5’-CAAAAGAACTCAGCCAAGTG-3’ |
|  | R :5’- TGGCGTCGGAACTGCAAAG-3’ |
| *N-cadherin* | F :5’-ACAGTGGCCACCTACAAAGG-3’ |
|  | R: 3’-CCGAGATGGGGTTGATAATG-5’ |
| *fibronectin* | F :5’-CAGTGGGAGACCTCGAGAAG-3’ |
|  | R :3’-TCCCTCGGAACATCAGAAAC-5’ |
| *vimentin* | F :5’-GAGAACTTTGCCGTTGAAGC -3’ |
|  | R :3’- GCTTCCTGTAGGTGGCAATC-5’ |
| *Snail1* | F: 5’-TTCTTCTGCGCTACTGCTGCG-3’ |
|  | R:5’GGGCAGGTATGGAGAGGAAGA-3’ |
| *Twist1* | F: 5’AGCTGAGCAAGATTCAGACCCTC-3’ |
|  | R: 5’-CCGTCTGGGAATCACTGTC-3’ |
| *Zeb1* | F: 5’-CTGAAGAGGACCAGAGGCAG-3’ |
|  | R: 5’-CCCAGAACTGCGTCACATGTC-3’ |
| *GAPDH* | F: 5’TGGTATCGTGGAAGGACTCATGAC-3’ |
|  | R: 5’-ATGCCAGTGAGCTTCCCGTTCAGC-3’ |
