# Supplementary figures and images for: Evidence that GTP-binding domain but not catalytic domain of transglutaminase 2 is essential for epithelial-to-mesenchymal transition in mammary epithelial cells
Source: Breast Cancer Res. 2012 Jan 6;14(1):R4. doi: 10.1186/bcr3085 (PMC3496119; doi:10.1186/bcr3085)

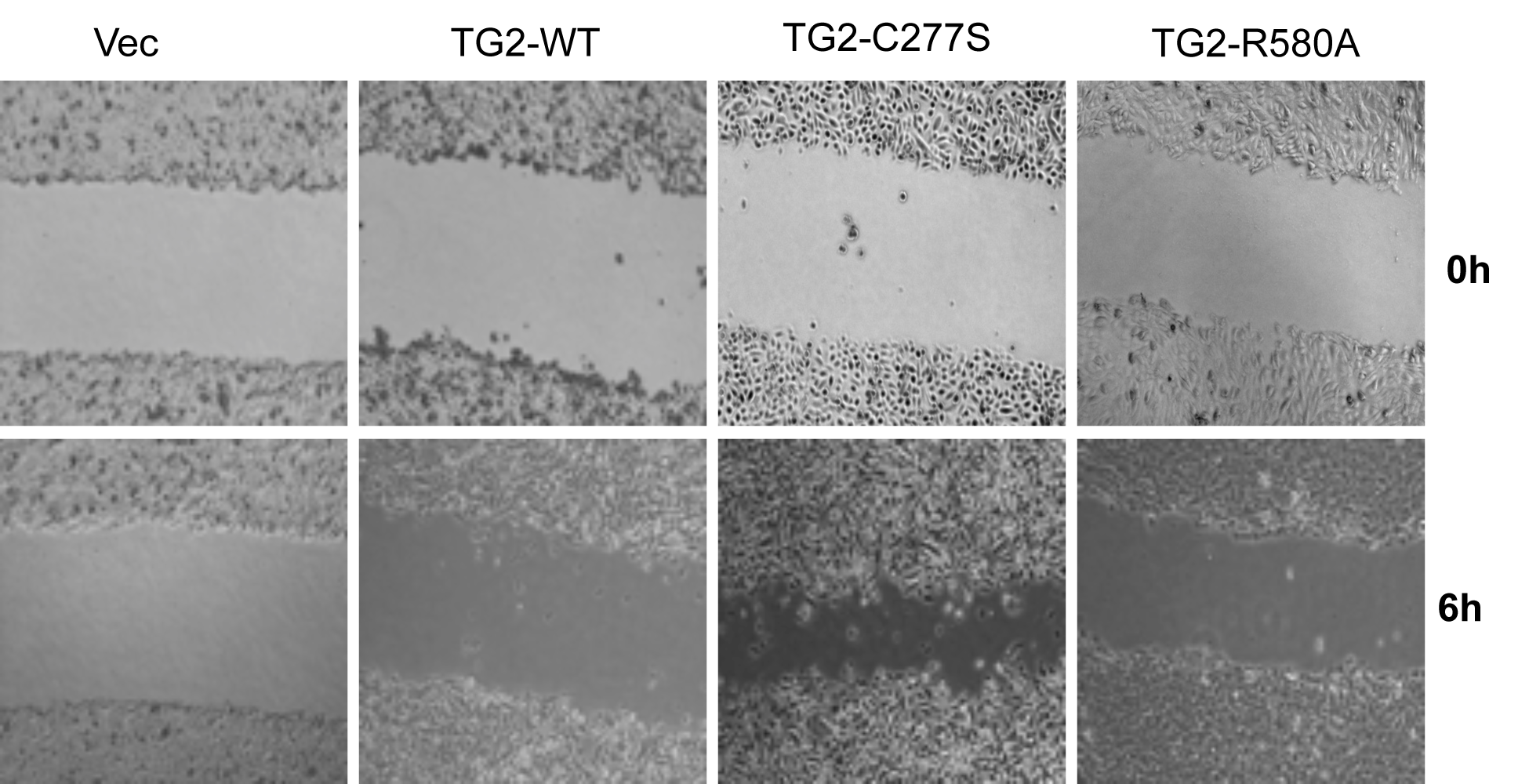

Supplement: Additional file 5 — Supplementary Figure 1Effect of various transglutaminase 2 constructs on cell motility. The wound-healing assay with indicated MCF10A sublines was performed. Cell cultures were photographed at 0 and 6 hours after wounding. [file bcr3085-S5.TIFF]

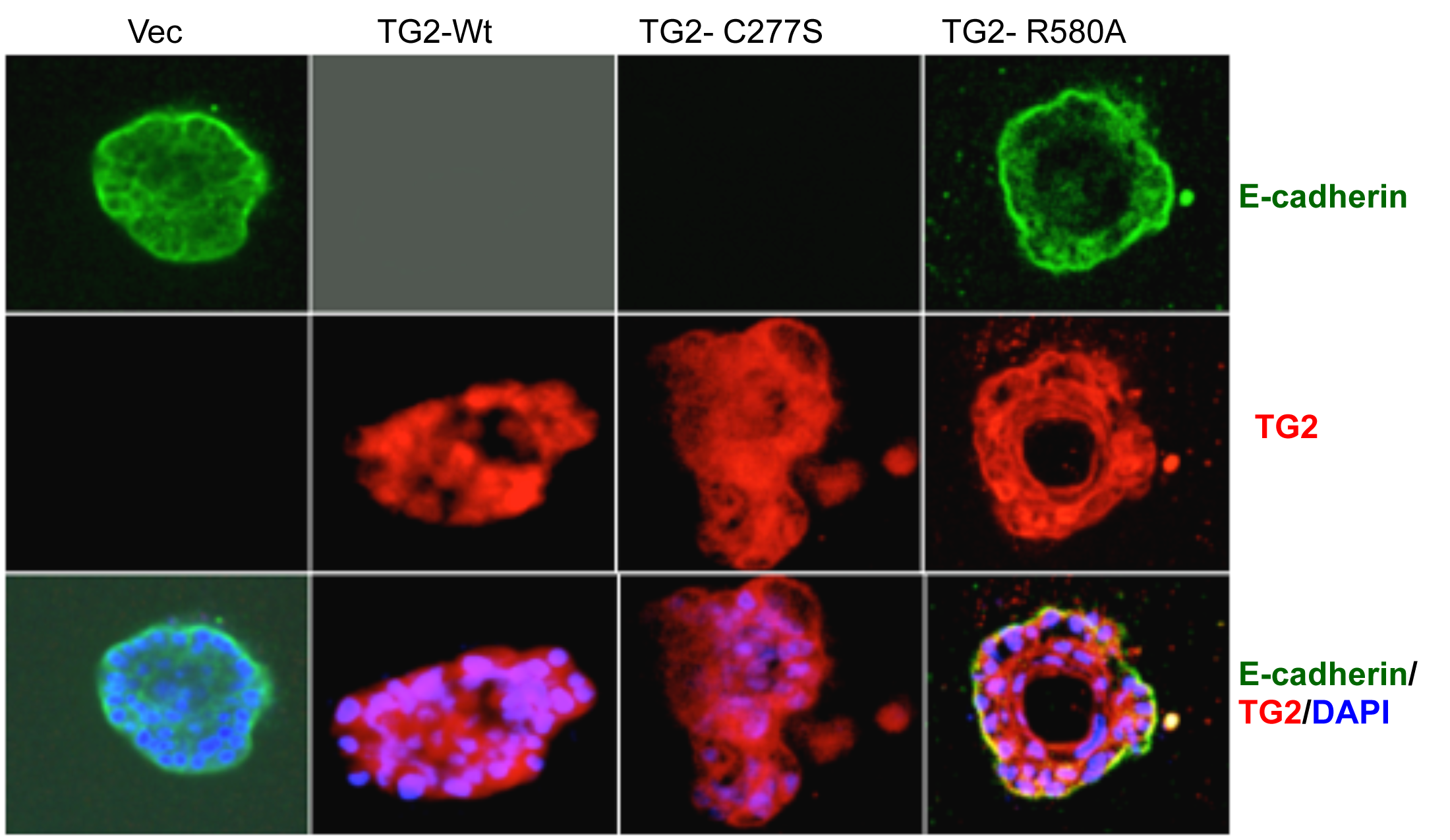

Supplement: Additional file 6 — Supplementary Figure 2Effect of various transglutaminase 2 constructs on acinar structure assembly. MCF10A cells stably transfected with indicated construct of transglutaminase 2 (TG2) were cultured in Matrigel-coated chambers for 12 days and immunostained for E-cadherin (green), TG2 (red) and 4',6-diamidino-2-phenylindole (blue). Representative images from two independent experiments with similar results are shown. Original magnification ×20. [file bcr3085-S6.TIFF]

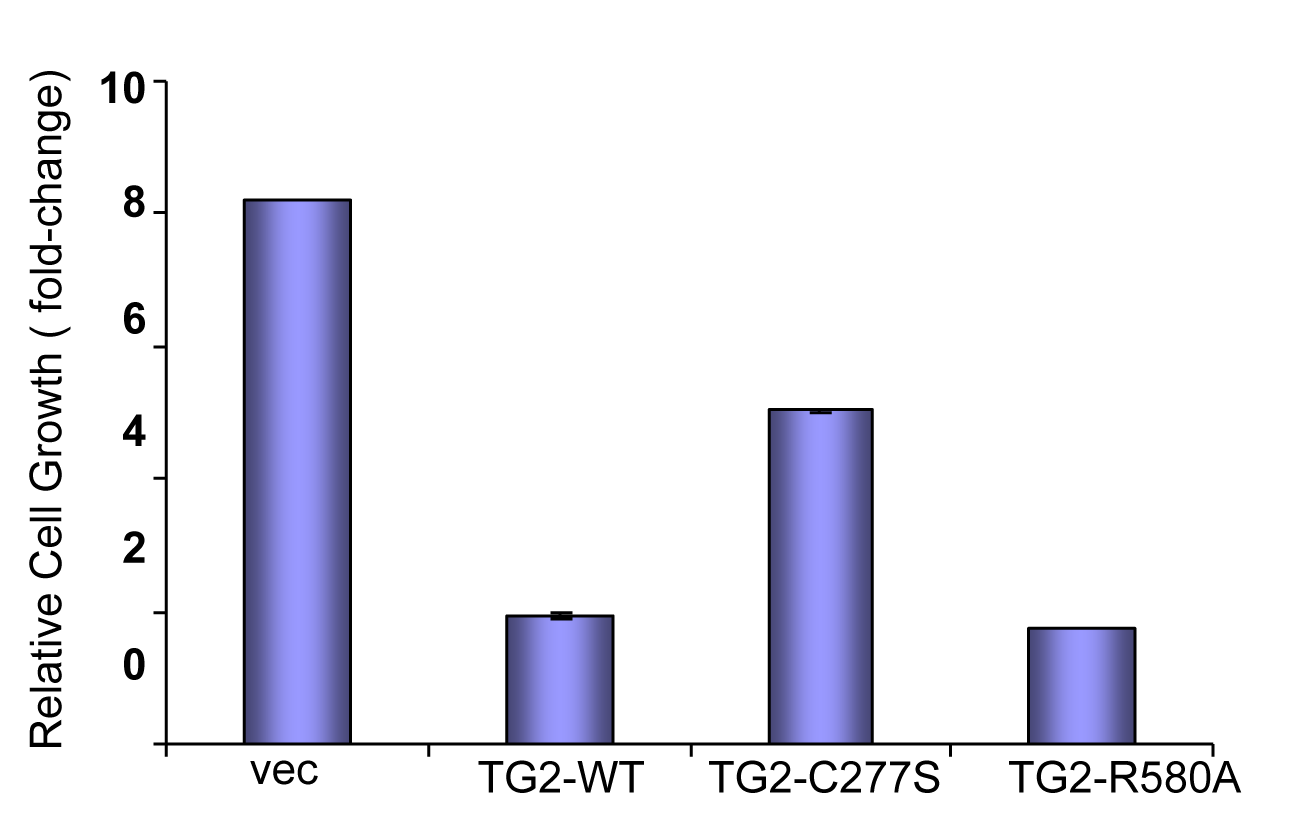

Supplement: Additional file 7 — Supplementary Figure 3Effect of various transglutaminase 2 constructs on MCF10A cell growth. Cells (n = 2,000) expressing indicated form of transglutaminase 2 (TG2) were cultured in quadruplicate in a 96-well plate, and the number of cells in each well was determined after 72 hours of culture. [file bcr3085-S7.TIFF]
